# Thermodynamics of Highly Interacting Blend PCHMA/dPS by TOF-SANS

**DOI:** 10.1021/acs.macromol.3c00511

**Published:** 2023-07-13

**Authors:** William N. Sharratt, Yutaka Aoki, Sebastian Pont, Dale Seddon, Charles Dewhurst, Lionel Porcar, Nigel Clarke, João T. Cabral

**Affiliations:** † Department of Chemical Engineering, Imperial College London, London SW7 2AZ, U.K.; ‡ Institut Laue Langevin, 71 Avenue des Martyrs, 38000 Grenoble, France; § Department of Physics, The University of Sheffield, Sheffield S10 2TN, U.K.

## Abstract

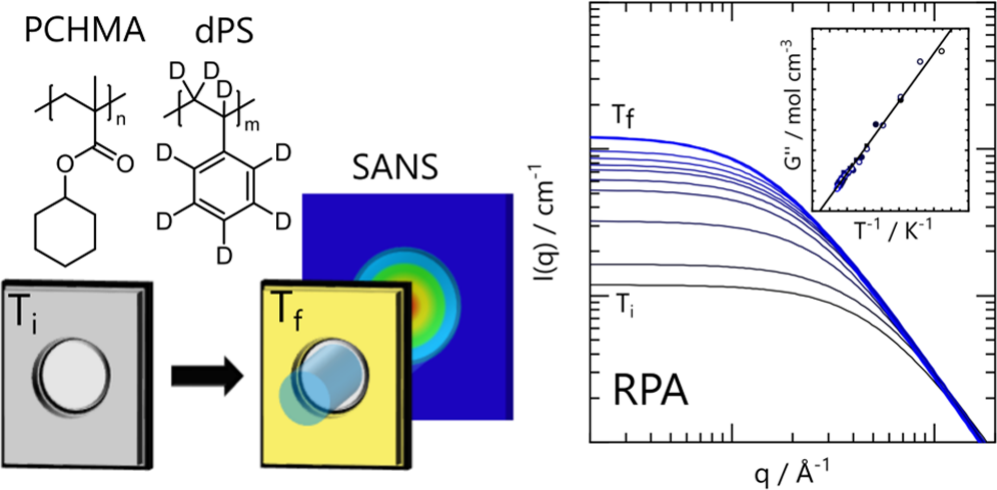

We investigate the thermodynamics of a highly interacting blend of poly(cyclohexyl methacrylate)/deuterated poly(styrene) (PCHMA/dPS) with small-angle neutron scattering (SANS). This system is experimentally challenging due to the proximity of the blend phase boundary (>200 °C) and degradation temperatures. To achieve the large wavenumber *q*-range and flux required for kinetic experiments, we employ a SANS diffractometer in time-of-flight (TOF) mode at a reactor source and ancillary microscopy, calorimetry, and thermal gravimetric analysis. Isothermal SANS data are well described by random-phase approximation (RPA), yielding the second derivative of the free energy of mixing (*G*″), the effective interaction (χ̅) parameter, and extrapolated spinodal temperatures. Instead of the Cahn–Hilliard–Cook (CHC) framework, temperature (*T*)-jump experiments within the one-phase region are found to be well described by the RPA at all temperatures away from the glass transition temperature, providing effectively near-equilibrium results. We employ CHC theory to estimate the blend mobility and *G*″(*T*) conditions where such an approximation holds. TOF-SANS is then used to precisely resolve *G*″(*T*) and χ̅(*T*) during *T*-jumps in intervals of a few seconds and overall timescales of a few minutes. PCHMA/dPS emerges as a highly interacting partially miscible blend, with a steep dependence of *G*″(*T*) [mol/cm^3^] = −0.00228 + 1.1821/*T* [K], which we benchmark against previously reported highly interacting lower critical solution temperature (LCST) polymer blends.

## Introduction

Understanding polymer blend thermodynamics and the roles of molecular architecture and specific interactions is required for the predictive design and fabrication of homogeneous and multiphase polymeric materials,^1−4^ with applications ranging from tissue engineering and organic photovoltaics to membrane technologies.^5−7^ Partially miscible lower critical solution temperature (LCST)-type blends are of particular interest, as demixing can be induced upon heating the blend into an unstable region, and the resulting structure can be arrested by rapid cooling below the glass transition temperature (*T*_g_). Partially miscible blends can be classified as ‘highly interacting’ when the interaction parameter χ changes steeply with the temperature near the stability boundaries,^4^ meaning that small demixed phases can be accessed with modest thermal quenches, which is attractive for material design.

In this paper, we examine the component interactions in the LCST blend of poly(cyclohexyl methacrylate)/deuterated poly(styrene) (PCHMA/dPS), previously investigated by optical microscopy and calorimetry,^8−15^ scanning electron microscopy and infrared spectroscopy,^14^ solid-state NMR,^15^ and rheology.^12^ The blend has been reported to phase-separate upon heating to 220–300 °C, depending on (PS) tacticity and molecular mass. We select this blend for investigation owing to the large accessible one-phase temperature range and the proximity of the glass transition temperatures (*T*_g_) of its constituents, yielding a relative dynamic ‘symmetry’. Further, recent correlations derived from Lipson and co-workers’ lattice-based equation of state suggest that this blend (with parameter *g* ∼ 1) could exhibit strongly temperature-dependent interactions.^16^ We employ small-angle neutron scattering (SANS) to resolve the blend thermodynamics within the single-phase region. Given the proximity of the blend’s phase boundaries to the degradation temperatures of its constituent polymers (>200 °C), we perform our experiments in time-of-flight (TOF) SANS on the D33 diffractometer at the Institut Laue Langevin (ILL), configured to simultaneously provide a large wavenumber (*q*) window and high neutron flux, as required for time-resolved experiments within timescales of seconds.

The single-phase region for this blend extends well beyond component degradation temperatures (>200 °C), and so we first establish accessible measurement temperature and timescales by thermal gravimetric analysis (TGA) before determining the temperature-composition phase boundary of the blend. We then select a near-critical composition (50/50 w/w) to elucidate the temperature dependence of the second derivative of the free energy of mixing (*G*″) and the effective interaction parameter (χ̅). Given the blends’ propensity to degrade at elevated temperatures close to the phase boundary, we utilize temperature-jump experiments within the single-phase region to demonstrate an approach to precisely determine the temperature of thermodynamic interactions across a wide temperature range. We employed de Gennes’ random-phase approximation (RPA)^17^ and the Cahn–Hilliard–Cook (CHC) framework^18−20^ to rationalize the experimental data and establish the validity of our nonequilibrium approach. Finally, we benchmark this blend with other well-known highly interacting blends in terms of *G*″(*T*) or equivalently χ̅(*T*) in the vicinity of the spinodal line.

## Experimental Section

### Polymer Mixtures

**Figure 1:**
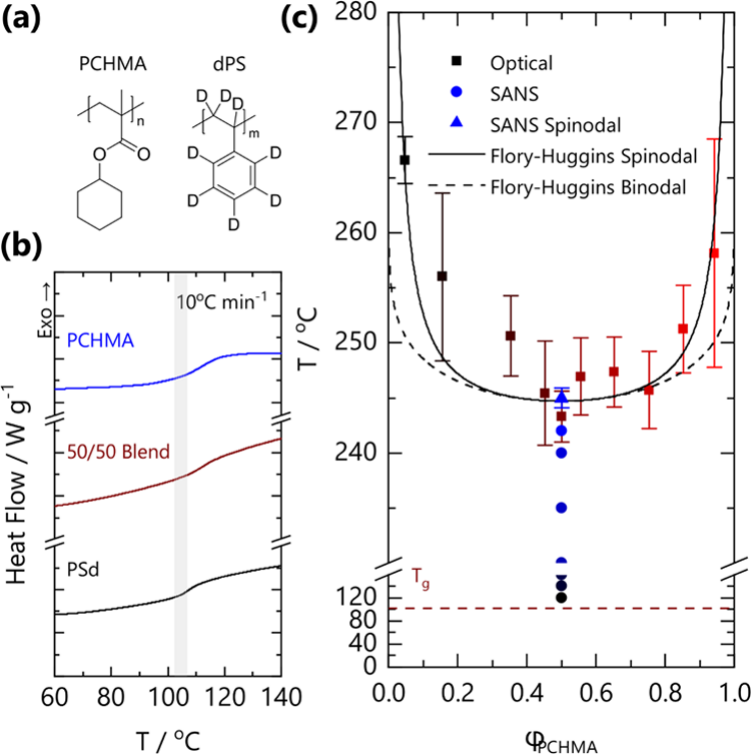
(a) Chemical structures of PCHMA and dPS. (b) DSC traces for PCHMA, dPS, and a 50/50 blend. Curves are shifted for clarity. The onset of the glass transition is indicated with the gray bar of ∼101–106 °C for the pure components and blend. (c) Phase diagram for PCHMA/dPS blend films. Optical data (■) correspond to *n* ≥ 3 independent measurements and maximum errors. Single-phase SANS measurements (blue solid circle) and the (extrapolated) spinodal temperature (blue solid triangle) are consistent with the optical cloud point curve. The solid and dashed lines are the calculated spinodal and binodal curves from Flory–Huggins theory for a symmetric blend with *N*_ave_ = 1706 and χ_FH_ = 0.139 – 71.2/*T*, obtained from SANS measurements.

Perdeuterated polystyrene (dPS) and poly(cyclohexyl methacrylate) (PCHMA) were purchased from Polymer Source Inc. and used as supplied. Key characteristics of the polymer samples used are summarized in Table 1, and the monomer chemical structures are shown in Figure 1a.

**Table 1:** Polymer Sample Characteristics: Weight-Average Molecular Mass ⟨*M*⟩_w_ and Polydispersity ⟨*M*⟩_w_/⟨*M*⟩*_n_*, the Mass of Each Repeat Unit, *m*, Polymerization Index, *N*, and Glass Transition Temperature, *T*_g_, Measured by DSC at 10 °C min^–1^

	⟨*M*⟩_w_		*m*	*N*	*T*_g_
	[kg mol^–1^]		[g mol^–1^]		[°C]
PCHMA	287	1.14	168.2	1496	101.6
dPS	253	1.15	112.2	1961	100.6

### Film Preparation

PCHMA/dPS films were prepared from 10% mass/volume solutions in tetrahydrofuran (THF, purity ≥99.7% unstabilised high-performance liquid chromatography (HPLC) grade, VWR). For SANS measurements, the solutions were drop cast onto glass coverslips (19 mm diameter, VWR), and the solvent was allowed to evaporate under ambient conditions for 48 h, yielding films of ∼100–150 μm thickness. For optical measurements, 1% g/cm^3^ solutions were drop cast directly onto silicon wafers, previously cleaned by ultraviolet (UV-ozone) (Novascan PSD) exposure for 5 min, resulting in ∼1 μm films. All samples were placed under vacuum (100 mbar) for one week while gradually increasing the temperature up to 150 °C, above the *T*_g_ of both components. Blend compositions were prepared by mass and then converted into volumetric ratios using the pure component densities.^21^

### Small-Angle Neutron Scattering

SANS experiments were performed at ILL using two diffractometers: D33, operating in time-of-flight (TOF) mode, and D22 in monochromatic mode (data reported in the Supporting Information (SI)). Details of samples and conditions measured on each diffractometer are tabulated in SI Table S1.

The D33 diffractometer was configured with sample-to-detector distances *D*_s–d1_ = 13.4 m for the rear detector and *D*_s–d2_ = 6 m for the enclosing 4-panel front detector bank.^22^ A polychromatic beam with neutron cutoff wavelength λ of 14 Å, yielding a usable TOF λ range of 1.5–12 Å, a wide Δλ/λ = 16.4% providing a *q*_min_ = 0.0017 Å^–1^, and a large momentum transfer window 0.0017 < *q* < 0.6 Å^–1^ in a single shot, with dynamic range *q*_max_/*q*_min_ ∼ 350, with no loss in flux (compared to equivalent and standard monochromatic configuration λ = 6 Å and Δλ/λ = 10%, *cf.* Fig. 2 in ref (22)), where  and θ is the scattering angle. The large Δλ/λ is acceptable for our measurements since a low-q resolution is required to characterize the scattering profiles of blends in the one-phase region. A custom-made brass experimental cell^23^ consisting of two thermally controlled brass blocks and a mechanical actuator that carried the sample from one (preheating) block to another (the ‘experimental block’), with quartz windows and a 45° exit cone, was employed.

In isothermal measurements, a film was wrapped in thin aluminum foil and first loaded into the preheating block at 120 °C and then transferred into the experimental block, whose temperature was gradually increased to the desired measurement temperature, and the sample was allowed to equilibrate. Temperature steps of 20 °C near *T*_g_ and 2 °C near the phase boundary were sampled. SANS acquisition times ranged from 60 min (10 min × 6) near *T*_g_ to 9 min (1.5 min × 6) closer to the phase boundary; thermal equilibration was verified by the invariance of the SANS data during the measurement within experimental uncertainty. Spectra from a 1 mm thick, hot-pressed PCHMA specimen, as well as from the empty cell (aluminum foil and quartz windows), and the blocked beam were acquired for 30 min, providing estimates for incoherent scattering of the blend (*I*_inc,PCHMA_ = 0.514 cm^–1^), empty cell, and electronic background.

For temperature-jump (*T-*jump) measurements, blend films were loaded into the preheating block at *T*_i_ and moved into the experimental block, also preheated to *T*_i_. An initial (reference) transmission and scattering measurement was acquired. The film was returned to the preheating block, and the temperature of the measurement block was raised to *T*_f_. SANS acquisition was then automatically triggered by the entrance of the film into the measurement block at *T*_f_, and profiles were acquired in 10 s intervals.

The scattering data were reduced and calibrated, and the contribution from the empty cell was subtracted using GRASP.^24^ The self-consistency between sample thickness, neutron transmission, and incoherent background intensity was verified to ensure accurate data calibration (as detailed in the Supporting Information, Table S1). The coherent scattering profile was then obtained by subtraction of the appropriate volume fraction of the incoherent contribution of PCHMA.

### Optical Cloud Point Measurements

Films cast directly onto silicon wafers were mounted on a thermal stage and imaged with a reflection microscope (Olympus BX41), CMOS camera (Basler acA2000-165 μm), and long-working distance objective (Olympus LMPLFLN 50X). The film surface temperature was monitored and calibrated measured with a K-type thermocouple and data logger (Pico TC-08) during the temperature ramp. To minimize the degradation of the polymers, films were heated rapidly to a fixed temperature below the phase boundary (200 °C) before heating at a rate of 10 °C/min, and images were acquired every 2 s.

### Atomic Force Microscopy (AFM)

Selected films were also examined by atomic force microscopy (AFM) using a Bruker Innova microscope in tapping mode at 0.2 Hz with Si tips (MPP-11100-W, Bruker). Supported blend films on silicon wafers were annealed for brief periods of time and rapidly quenched with a large thermal mass of cold stainless steel prior to imaging in order to corroborate the location of the phase boundaries.

### Thermal Gravimetric Analysis

The thermal degradation of blend films was assessed by thermal gravimetric analysis (TGA, NZ STA Jupyter). Samples were rapidly (5 °C min^–1^) heated from above the *T*_g_ of the film to 240 °C and held isothermally for 60 min.

### Differential Scanning Calorimetry

*T*_g_ of pure polymer components and blends were determined by differential scanning calorimetry (DSC, TA Instruments Q2000). Samples of mass 3–12 mg were sealed in hermetic aluminum pans, heated to 155 °C to erase thermal history, and rapidly quenched to 25 °C before heating at 5, 10, and 20 °C min^–1^ under a nitrogen atmosphere; *T*_g_ values were estimated by the onset method and are tabulated in SI Table S2.

## Results and Discussion

Components PS and PCHMA exhibit a nearly identical *T*_g_ of 101 °C, as shown in Figure 1b, while the glass transition of blends is slightly broader and the *T*_g_ appears slightly shifted to a higher temperature.^12,15^ DSC profiles acquired at distinct rates (5–20 °C min^–1^) are provided in SI Figure S2, and an increase in (apparent) *T*_g_ with the rate was observed, as expected. Figure 1c shows the cloud point data obtained by optical microscopy as a function of blend composition (ϕ_PCHMA_).

The optical phase boundary appears somewhat asymmetric, with the critical point shifted toward PCHMA and located at high temperatures (with the critical point at ∼244 °C), well above the ceiling temperature of the constituent polymers (>200 °C). This boundary is in broad agreement with previous reports.^8,12,15^ We interpret the uncertainty associated with these measurements as due to thermal degradation and the heating-rate dependence of cloud point estimates.

The binodal and spinodal lines are computed according to Flory–Huggins theory,^3,25−27^ which describes the data satisfactorily, within uncertainty, with a composition-independent interaction parameter χ_FH_ = *A* + *B*/*T* where *A* = 0.139 and *B* = −71.2 K. These values differ from those reported by Friedrich et al.^12^ (*A* = 0.022 and *B* = −10.759) who suggested a favorable comparison with previous interaction energy density estimates.^8,10^ While the experimentally measured cloud point curves broadly agree, we attribute the differences in χ(*T*) to large uncertainties in the optical detection of phase boundaries (ref (12) does not provide error bars) and limited data sets, resulting in the estimated χ(*T*) ≡ *A* + *B*/*T* parameters not being single-valued, as illustrated in SI Figure S3. Below, we report SANS data in the one-phase region, obtaining χ(*T*) across a wide range of temperatures, which resolves this discrepancy, accounting for the current and previously reported phase boundaries. Isothermal SANS measurements for 50/50 blends, which reside in the single-phase region, are indicated in Figure 1c, alongside the extrapolated spinodal temperature, which is in good agreement with the optical cloud point curve.

**Figure 2:**
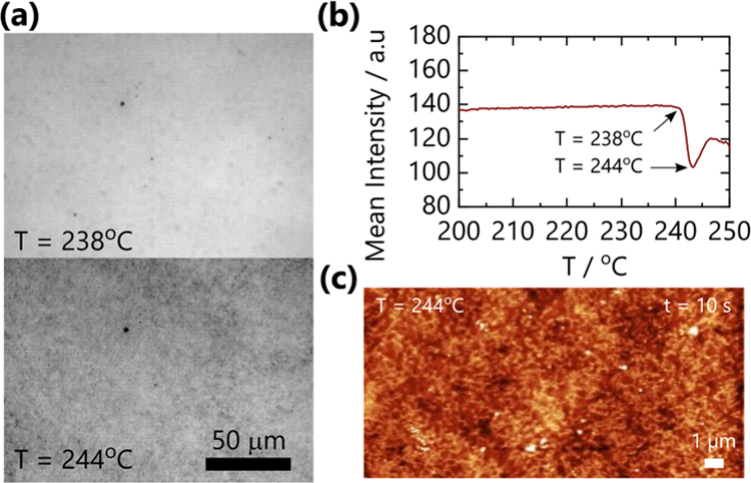
(a) Optical images of a 50/50 blend film at *T* = 238 °C and *T* = 244 °C show the appearance of small micron-sized domains and the shift in light intensity observed. (b) Mean pixel intensity from optical images of a 50/50 blend film during a 10° min^–1^ temperature ramp quantifies the observable shift in intensity. The observed intensity shifts prior to the appearance of micron-sized domains. (c) AFM image of an isothermally annealed 50/50 blend film at 244 °C for 10 s confirms the appearance of phase separation in the film with a length scale of ∼500 nm.

The location of the phase boundary was confirmed by optical microscopy and AFM measurements of specimens quenched in the two-phase region, as shown in Figure 2. Micron-sized domains can be observed optically, coarsening rapidly over time at 244 °C (Figure 2a). Tracing the mean pixel intensity profile over time, or temperature, during a ramp provides reasonable (upper) estimates of the onset of demixing, as shown in Figure 2b. Films annealed into the two-phase region and rapidly quenched below *T*_g_ exhibit nanoscale topography, depicted in Figure 2c, for a 50/50 blend annealed at 244 °C for 10 s, with a relatively large characteristic length scale of ∼500 nm, which increases rapidly with time and further annealing.

The isothermal SANS measurements for the binary polymer blends in the one-phase region were analyzed following established procedures. The coherent scattering intensity reads
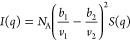
1where *N*_A_ is the Avogadro number, *b_i_* is the coherent scattering length of the monomer unit *i*, and *v_i_* is the monomer molar volume of unit *i*; we refer to PCHMA as species 1 and dPS as species 2, for which *b*_1_ = 18.22 fm, *v*_1_ = 152.9 cm^3^ mol^–1^, *b*_2_ = 106.54 fm, and *v*_2_ = 100.2 cm^3^ mol^–1^, which yield a contrast prefactor *N*_A_ (*b*_1_/*v*_1_ – *b*_2_/*v*_2_)^2^ = 5.37 × 10^–3^ cm^–4^ mol. The structure factor *S*(*q*) of the blend is expressed by de Gennes random-phase approximation (RPA)^17^ as
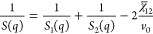
2where *S_i_*(*q*) (cm^3^ mol^–1^) is the partial structure factor of each component and χ̅_12_ is the effective interaction parameter of the blend. Taking component polydispersity into account^23,28^
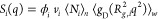
3where ϕ*_i_* is the volume fraction, *v*_0_ is a reference volume taken as , and ⟨*N_i_*⟩*_n_* is the number-average degree of polymerization of component *i*. The weight-average Debye form factor of the polymer chains is , where *x* ≡ *q*^2^⟨*R*_*g*_^2^⟩*_n_* and *h* = (*M*_w_/*M_n_* – 1)^−1^, and the *n*-average radius of gyration for a Gaussian coil ⟨*R_g_*⟩*_n_* ≡ (⟨*N*⟩*_n_a*^2^/6)^0.5^, where *a* is the segment length. In the forward scattering limit, *q* → 0, eq 2 becomes
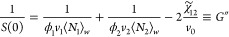
4yielding a direct measurement of the second derivative of the free energy with respect to composition, *G*″ ≡ ∂^2^Δ*G*_m_/∂ϕ^2^. Assuming Flory–Huggins theory, the interaction parameter χ_s_ at the spinodal is
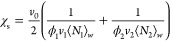
5and therefore, eq 4 can be expressed as
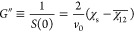
6providing a facile estimate of χ_12_ (which is assumed to be composition-independent).

**Figure 3:**
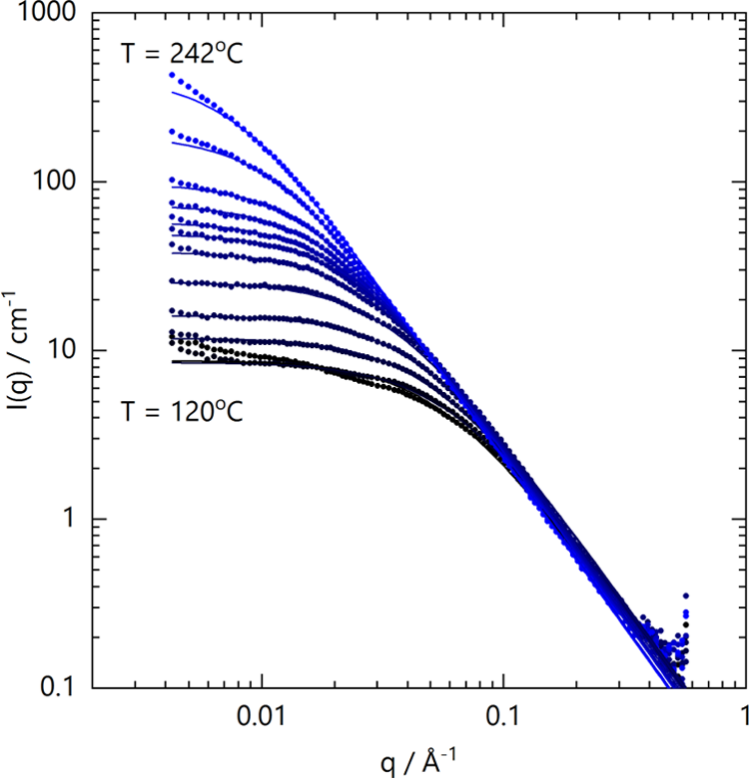
(a) Coherent scattering profiles for 50/50 PCHMA/dPS blends measured by TOF-SANS on the D33 diffractometer at *T* = 120, 140, 160, 180, 200, 210, 220, 225, 230, 235, 240, and 242 °C (black to blue filled circles). Solid lines are fits to the RPA (see text).

Experimentally measured *I*(*q*) for 50/50 PCHMA/dPS blends, as a function of temperature from 120 to 242 °C, acquired in TOF-SANS on D33, are shown in Figure 3. RPA describes all data satisfactorily, with two fitting parameters: χ̅_12_ and *a*_PCHMA_, as we fix the dPS segment length to *a*_dPS_ = 6.7 Å,^29^ in line with previous reports. Estimates for *a*_PCHMA_ and a separate discussion of the Kratky analysis of the scattering data are included in the Supporting Information (SI Figure S5). While the RPA describes all data within 160–242 °C, deviations at low *q* are found below 160 °C, which are attributed to the slow equilibration of long wavelength fluctuations.^23,30,31^ We have also attempted to acquire SANS data of the blend up to 260 °C, with progressively smaller acquisition times, in order to minimize degradation. We have separately determined the mass loss of the blend held isothermally at 240 °C by TGA in SI Figure S4, yielding a loss of ≲1 % mass over a period of ∼5 min, and all high-temperature measurements were thus restricted to times shorter than this. However, depolymerization is expected to change *M*_w_ and result in plasticising oligomeric and monomeric species (in addition to chemical transformations), which can considerably alter blend thermodynamics and SANS profiles. While for temperatures ≤242 °C, the SANS profiles were found to be stable across the whole *q*-range within measurement timescales, at 244 °C and above, the low*-q* scattering evolves with time, indicating the onset of demixing and/or degradation within measurement timescales.

**Figure 4:**
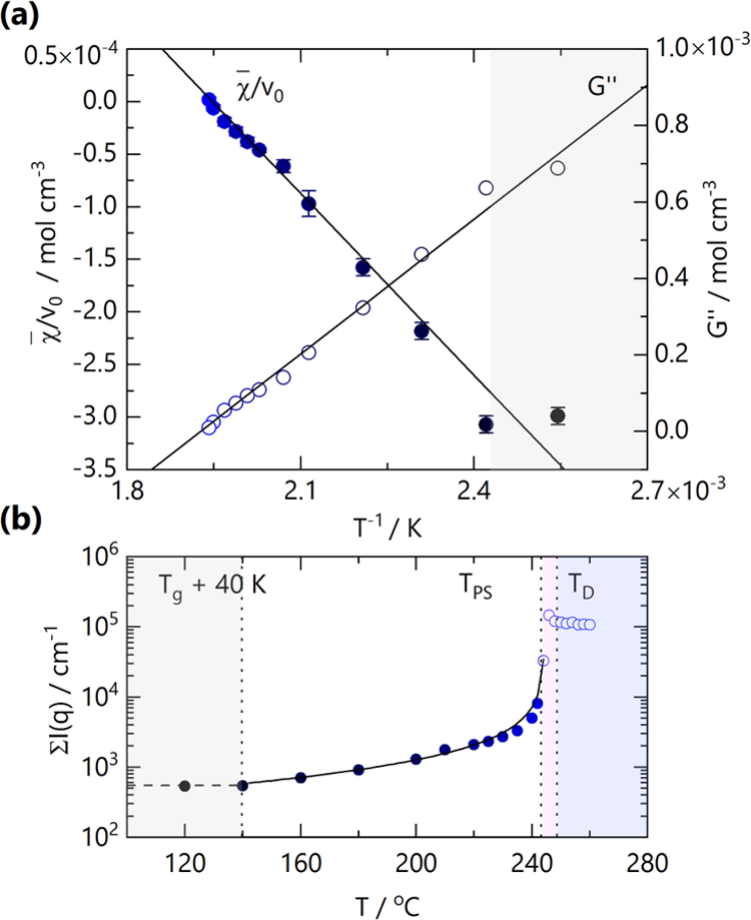
(a) Linear dependence of χ̅/*v*_0_ (filled circles, left axis) and *G*” (open circles, right) with inverse temperature, 1/*T*. Data points within ≈ 40 K of *T*_g_, indicated by the gray-shaded region, did not appear to fully equilibrate within measurement timescales and were thus not included in the linear fits. (b) Sum of coherent scattering profiles from Figure 3 at each measurement temperature. Open circles represent measured SANS profiles beyond the phase boundary. The RPA prediction of the total scattering (solid line) reasonably tracks the scattering intensity up to the phase boundary, where it begins to diverge. Shaded regions indicate *T*_g_ + 40 K (gray), phase separation *T*_PS_ (pink), and the onset of significant degradation, which causes a reduction in the sum of scattering intensity *T*_D_ (blue).

Figure 4a shows the temperature dependence of χ̅/*v*_0_ and *G*″, estimated from RPA fits to isothermal SANS data. As expected, these are proportional to 1/*T* at temperatures sufficiently above *T*_g_, specifically *T* > *T*_g_ + 40 K. Close to the *T*_g_, the low*-q* (and thus large wavelength) concentration fluctuations within the blend do not appear to equilibrate within measurement timescales, leading to RPA deviations at low *q*. This region is indicated by the gray-shaded area. Linear fits to the data yield χ̅/*v*_0_ = 0.00112 – 0.575/*T* and *G*″ = −0.00226 + 1.173/*T* mol cm^–3^, respectively. Extrapolation of χ̅/*v*_0_ to χ_s_®/*v*_0_ and *G*″ to 0 yields the spinodal temperature *T*_s_ = 245.0 ± 0.9 °C. This value agrees, within measurement uncertainty, with the observed location of the phase boundary (*T* ≈ 244 °C). With an estimated critical composition , our 50/50 w/w blend is slightly off-critical and would suggest that the observed phase separation could be nucleation and growth prior to spinodal decomposition. Analysis of the high-q region of the data via a Kratky analysis is presented in SI Figure S5, yielding asymptotic *I*(*q*)*q*^2^ ≈ 0.023–0.028 cm^–1^ Å^–2^ and estimates for the PCHMA segment length of *a*_PCHMA_ = 13.9 ± 0.6 Å.

We evaluate the total scattering intensity on the detector Σ*I*(*q*) (*i.e.*, the intensity sum across the whole q-range) at each temperature to define regions of behavior, as shown in Figure 4b. At the lower temperatures investigated, within *T*_g_ + 40 K, the blends do not appear to have reached thermal equilibrium, as shown by the gray shared area. Within *T* = 120–244 °C, the RPA describes well the sum of scattering intensity, as shown by the agreement between the (calculated) solid line and experimental data points. At higher temperatures, Σ*I*(*q*) first diverges to higher values, reflecting increased forward scattering at the phase separation temperature, *T*_PS_, before decreasing slightly, which we attribute to the onset of considerable degradation within measurement timescales.

SANS measurements of blends as a function of composition are presented in SI Figure S6, yielding *G*″(*T*, ϕ) estimates. At a fixed temperature within the single-phase region, *G*″ appears to follow a shallow, parabolic concentration dependence. For simplicity, however, in Figure 1, we compute the phase boundaries with FH theory and a composition-independent χ, obtained from the χ̅/*v*_0_ linear (1/*T*) fit in Figure 4 with a reference volume *v*_0_ = 123.8 cm^3^ mol^–1^. This yields χ̅ = 0.139–71.2/*T*, which satisfactorily describes both the present and previously reported^12^ optical cloud point data, as well as the SANS stability boundary for the near-critical 50/50 PCHMA/dPS blend. In contrast, the value report by Friedrich et al. corresponds to a much shallower temperature dependence of χ̅/*v*_0_, which is not observed.

The limited time available for one-phase SANS measurements approaching the phase boundaries at such elevated temperatures (bound by commensurate degradation temperatures and timescales) led us to consider the feasibility of quasi-equilibrium SANS measurements during temperature ramps or jumps. The diffractometer D33 was thus configured in TOF-SANS mode, using a broad neutron wavelength distribution and low wavelength resolution Δλ/λ, and in return, a wide dynamic *q*-range and high flux, as described above. In this way, short measurement timescales (10 s) were attainable in the one-phase region with a single (polychromatic) spectrometer configuration (*i.e.*, without requiring several sample-to-detector distances or wavelength changes), yielding acceptable statistics for RPA fits from which *G*″ can be readily estimated. We note that kinetic SANS experiments of polymer blend demixing are routinely carried out at such timescales (5–15 s per spectrum), but the characteristic scattering intensities of phase separating blends (100–100000 cm^–1^) and generally several orders of magnitude greater than those of one-phase blends (10–100 cm^–1^) of near-symmetric hydrogenated/deuterated systems.^23,32,33^ The lower *q* resolution of our TOF-SANS measurements is acceptable for the purpose of characterizing the slowly varying Lorentzian profile that describes one-phase polymer blends in the RPA framework (but would not be appropriate to resolve, for instance, sharp structural peaks).

**Figure 5:**
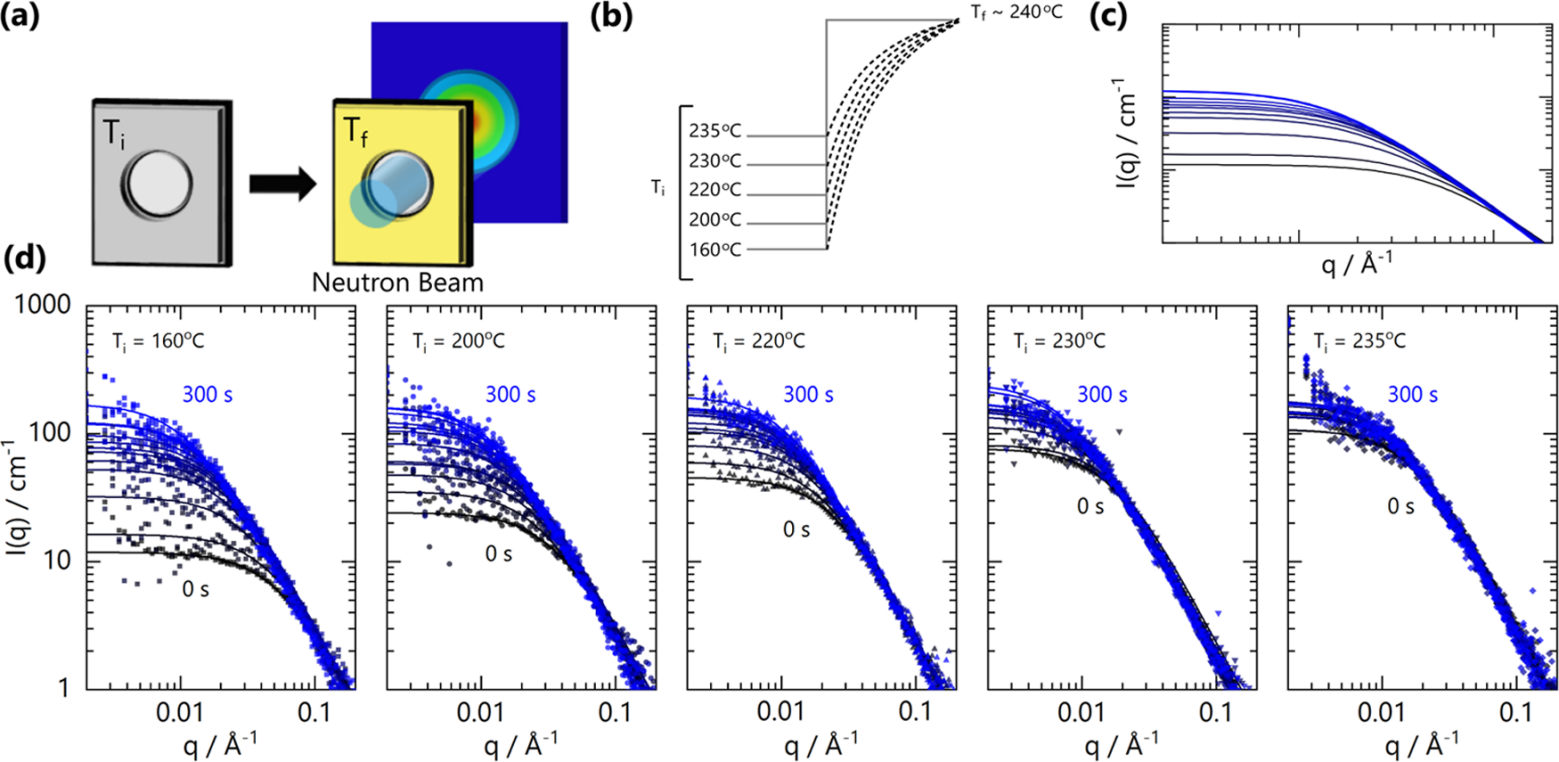
(a) Schematic of the experimental setup for temperature-jump SANS experiments, where blend films are transferred from a preheating block at *T*_i_ into a measurement block at *T*_f_. The setup enables ‘dynamic’ measurements of blend thermodynamics. (b) Schematic temperature profiles for jumps from various *T*_i_ to a fixed *T*_i_ ∼ 240 °C. (c) Calculate RPA profiles for a ‘slow’ temperature jump (with respect to blend mobility *M*), allowing for near-equilibrium *S*(*q*) measurement at varying temperatures. (d) SANS data acquired in 10 s intervals (from 0 to 300 s) during the temperature jumps illustrated in panel (b). Lines are RPA fits.

We illustrate our experimental approach schematically in Figure 5a–c. Films were preheated to *T*_i_ and transferred to a measurement block at *T*_f_. In the experiments shown here, we selected a fixed *T*_f_ = 240 °C and systematically varied *T*_i_ from 160 to 235 °C. During the rapid heating of the film from *T*_i_ to *T*_f_, time-resolved SANS measurements were acquired, with 10 s resolution. With the experimental setup and material proprieties employed (brass blocks and sample carrier), the sample follows a relatively ‘slow jump’ profile from *T*_i_ to *T*_f_ in ∼100 s. Specifically, equilibration times range from ≃60 to 285 s, depending on Δ*T* ≡ *T*_f_ – *T*_i_, which ranges from 5 to 80 °C. The estimated temperature profile is discussed in SI Figure S7. Figure 5d shows five representative temperature jumps from distinct *T*_i_ values, with 10 s time resolution during the jump. Within the time range evaluated, no obvious signs of thermal degradation (*e.g.*, change in high-*q* RPA profiles or mass loss) were observed. While we expected CHC to describe the temperature-jump scattering data,^30^ we found instead that RPA could describe all our time-resolved data. An apparent *G*″, and thus χ̅/*v*_0_, can be readily extracted from each profile, yielding *G*″ as a function of time and temperature, as discussed below.

Temperature-jump experiments in polymer blends are generally interpreted within the framework of Cahn–Hilliard–Cook (CHC) theory,^18−20^ which describes the evolution of the concentration fluctuation spectrum following a quench. While many studies apply CHC to describe the earliest stages of spinodal decomposition,^4^ following a temperature jump inside the spinodal line, CHC theory applies (arguably better) to one-phase jumps,^30^ describing the equilibration between two one-phase states. Incorporating the RPA into CHC theory,^30,34−36^ the evolution of the structure factor *S*(*q*,*t*) of a polymer blend can be written as
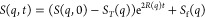
7where *S*(*q*, 0) ≡ *S*_i_ is the initial structure factor at *t* = 0, and *S_T_*(*q*) is the final structure factor, following the jump to *T*_f_ after equilibration (in a jump into the spinodal region *S*_*T*_(*q*) becomes a virtual structure factor, which cannot be experimentally measured). In the linearized theory, the *q*-dependent rate of change (growth or decay) *R*(*q*) of concentration fluctuation amplitudes is given by
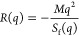
8where *M* is a diffusional mobility term related to the mutual diffusion coefficient of the constituent polymers and is strongly temperature-dependent.

**Figure 6:**
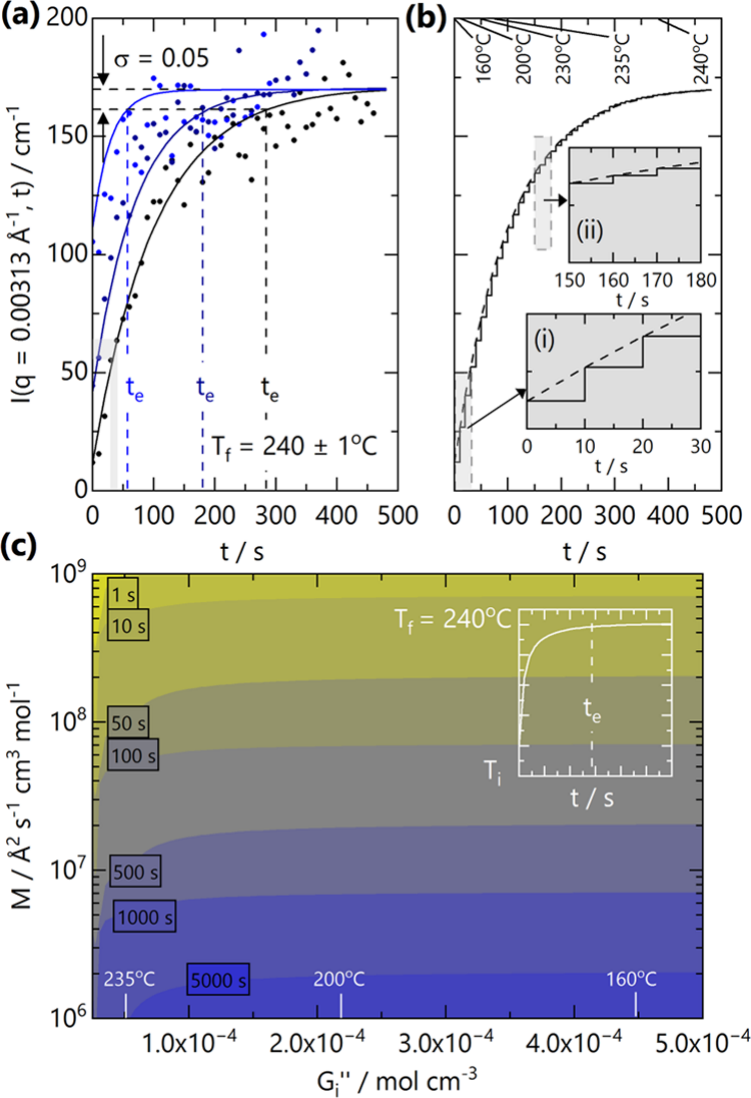
(a) Scattering intensity evolution with time at a fixed (low) *q* = 0.00313 Å^–1^ following temperature jumps from *T*_i_ = 160 °C (black), 220 °C (navy blue), and 235 °C (blue) to *T*_f_ = 240 °C. Lines are descriptive fits to CHC theory. Estimated equilibrium times *t*_e_ are indicated by the vertical dashed lines, computed from a proximity parameter σ (see text). The gray-shaded region indicates the largest variation in *I*(*q*) during a SANS timestep (10 s). (b) At finite heating rates and rapid equilibration of blends, temperature jumps from *T*_i_ to *T*_f_ can be considered as a series of “instantaneous” jumps and isothermal plateaus. The dashed line is a CHC guide to the eye for a jump from *T*_i_ = 160 °C, and the solid line illustrates a series of “instantaneous” jumps. Insets describe the relative magnitude of *I*(*q*,*t*) changes at the early (i) 0–30 s and intermediate times (ii) 150–180 s during the jump. (c) Color map of the effect of blend mobility *M* and initial *G*_i_^″^ on effective equilibration time *t*_e_ of the blend, as calculated from eq 10, for a fixed *T*_f_ = 240 °C. Along the *G*_i_^″^ axis, we marked corresponding *T*_i_ temperatures for this system. A fixed proximity parameter σ = 0.05 and a low*-q* limit (0.003 Å^–1^) were selected. The inset illustrates the temperature profile from *T*_i_ to *T*_f_ = 240 °C.

The evolution of the scattering intensity following a one-phase jump at a fixed q-value is illustrated in Figure 6a. In order to estimate an effective equilibration time for *S*(*q*,*t*), assuming an instantaneous temperature jump from *T*_i_ to *T*_f_, we introduce a ‘proximity parameter’ σ, which defines how close *S*(*q*,*t*) is to the asymptotic equilibrium value *S*_f_(*q*). In practical terms, a blend can be considered to be effectively ‘equilibrated’ once *S*(*q*,*t*) reaches a fraction (1 – σ) of *S*_f_(*q*) at experimental time *t*_e_, such that *S*(*q*,*t*_e_) = *S*_f_(*q*) (1 – σ). *t*_e_ can be expressed as
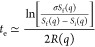
9as detailed in the SI. The parameter σ can formally take values from 0 to 1, but we select σ = 0.05, which means within 5% of the asymptotic value *S*_f_(*q*), based on typical uncertainties in SANS beamlines. We consider this to be reasonable for our measurements as short acquisition times result in inevitable scatter and greater uncertainty in the data. In the RPA framework, the forward scattering yields a measure of the blend thermodynamics *S*(*q* → 0) ≡ 1/*G*″. As the large wavelength, low*-q* concentration fluctuations take the longest time to equilibrate, we approximate eq 9 in this limit, yielding
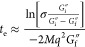
10where *G*_i_^″^ and *G*_f_^″^ are the *G*″ values at the initial, *T*_i_, and final, *T*_f_, temperatures of the jump.

Figure 6a illustrates the evolution of scattering intensity at a fixed *q*-value of 0.00313 Å^–1^ for temperature jumps from *T*_i_ = 160, 220, and 235 °C to *T*_f_ = 240 °C. In our experiments, however, we note that SANS profiles are adequately fitted to RPA at each acquisition timestep (10 s), expected for isothermal instead of temperature-jump experiments. It is expected that, for sufficiently high *M*, the evolution of *S*(*q*) with time (and temperature) can instead be described by a series of near-isothermal steps, which equilibrate ‘instantaneously,’ *i.e.*, faster compared to measurement timescales, effectively tracking the temperature profile. Figure 6b illustrates this stepwise evolution for a temperature jump from *T*_i_ = 160 °C to *T*_f_ = 240 °C, the largest jump investigated. Provided that sufficient data statistics are attained within an acquisition period (here Δ*t* = 10 s) to resolve the *S*(*q*) profile with sufficient accuracy, a short Δ*t* should ensure that the scattering data encompass a narrow Δ*T* and can thus characterize *G*″(*T*) at a well-defined temperature. The effect of ‘slow’ temperature jumps has indeed been considered by several authors, including Binder and co-workers, who modeled the “influence of a continuous quenching procedure on the initial stages of spinodal decomposition,” recognizing the practical limitations of implementing ‘instantaneous’ temperature jumps.^37^

In order to estimate a lower boundary for the mobility parameter *M*, we consider the *I*(*q*) data in Figure 6a, assuming that it corresponds to a fast, or instantaneous, temperature jump. The apparent equilibration time *t*_e_ is indicated by the dashed vertical lines, corresponding to (1 – σ) *I*_f_(*q*) for each temperature jump (varying *T*_i_ and at fixed *T*_f_). The intensity profiles appear generally well described by CHC theory, eq 7, providing an estimation for *M*. The equilibration time criterion introduced above allows for a facile estimation of *t*_e_ from the experimental data directly. Alternatively, a prediction of *t*_e_ (in the forward scattering limit *q* → 0) can be readily made based on an accurate mobility *M* estimate,^4,38^*G*″(*T*), and the temperature jump Δ*T* ≡ *T*_f_ – *T*_i_, as illustrated in Figure 6c, for a selected *T*_f_ = 240 °C, mirroring our experiments. The (apparent) equilibration timescale *t*_e_ decreases, as expected, with quench depth Δ*T*: from 285 s at *T*_i_ = 160 °C to 59 s at *T*_i_ = 235 °C, a ∼5-fold decrease. From eq 10, the estimated *M* changes over this temperature range by an estimated ∼3.3-fold increase (1.6 × 10^7^ to 5.2 × 10^7^ Å^2^ s^–1^ cm^3^ mol^–1^), while *G*_i_^″^ evidently changes for the different *T*_i_. Estimation of *M* through eq 10 for arbitrary *T*_i_ and *T*_f_ can be made by substitution of their respective *G*_i_^″^ and *G*_f_^″^ values from *G*″(*T*) and experimentally determined *t*_e_ (Figure 6a). These high *M* values are not unexpected, given the high temperatures of the phase boundaries with respect to *T*_g_. The values are an order of magnitude greater than typical blend mobility values (∼10^6^ Å^2^ s^–1^),^4^ which reflects a typical temperature dependence for the viscosity or diffusion coefficient of blend constituents.

Figure 6c provides a visual representation of eq 10, where the color map indicates the expected *t*_e_ for a blend with a characteristic *M*, and *G*_i_^″^ (or equivalent *T*_i_), σ = 0.05 and with fixed *G*_f_^″^ = 2.59 × 10^–5^ mol cm^–3^ (*T*_f_ = 240 °C). Evidently, higher *M* leads to faster equilibration times, but the dependence on initial temperature, or *G*_i_^″^ ∝ 1/*T*, results in a more complex dependence. For larger temperature jumps, starting from higher *G*_i_^″^, *t*_e_ is predominantly governed by the blend mobility. As *G*_i_^″^ → *G*_f_^″^ and Δ*G*″ → 0, *t*_e_ decreases rapidly, given the proximity between final and initial states. Mobility *M* ≥ 10^7^ Å^2^ s^–1^ cm^3^ mol^–1^ is required for these blends to equilibrate within our maximum experimental timeframe (∼500 s).

**Figure 7:**
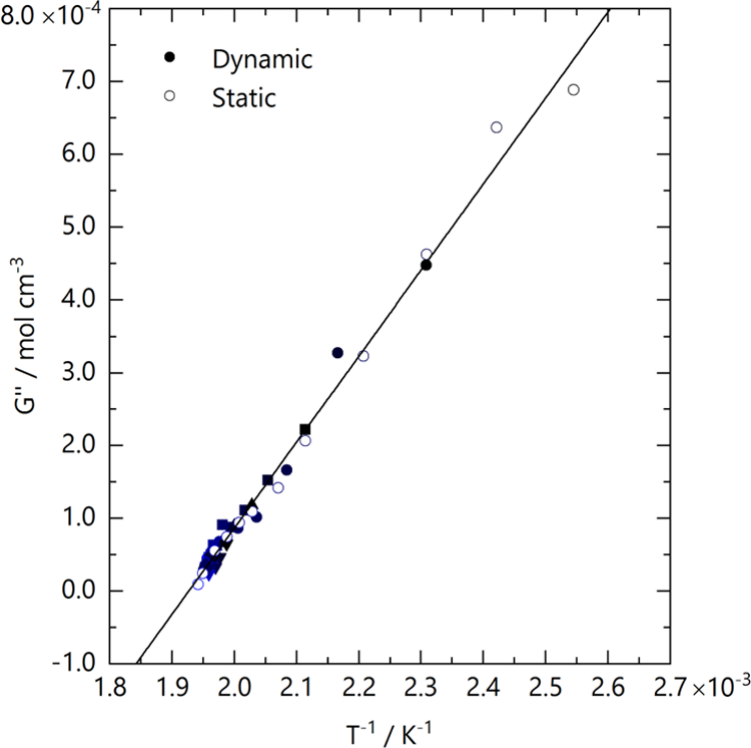
Comparison of *G*″ values extracted from static isothermal step profile measurements (Figure 3) and dynamic temperature-jump measurements (Figure 5d). The resulting combined data set yields a linear inverse temperature dependence for *G*″ = −0.00228 + 1.1821/*T*.

We next compare the series of *G*″(*T*) values estimated from the five temperature-jump experiments (from 10 s acquisitions), termed ‘dynamic,’ with the values determined by isothermal or ‘static’ measurements over comparatively longer times (1 h close to *T*_g_ to ∼10 min close to and above *T*_s_). These data are plotted in Figure 7, showing an excellent agreement between data sets and therefore supporting the quasi-equilibrium nature of the temperature-jump measurements in this high *M* system. Linear fitting of the ensemble of data yields a refined temperature dependence *G*″ = −0.00228 + 1.1821/*T*, particularly for temperatures close to the phase boundary, which are densely populated with data. Equivalently, this yields χ̅/*v*_0_ = 0.00115 – 0.591/*T* and *T*_s_ = 245.3 ± 0.9 °C. Under conditions of high *M* and noninstantaneous temperature jumps (≫*t*_e_), we conclude that such a quasi-isothermal approximation is not only appropriate for such measurements but provides a powerful and simple means to extract large data sets within relatively small timescales, able to fully characterize blend thermodynamics *G*″(*T*). Under high-temperature conditions, where thermal degradation becomes problematic, this approach seems particularly well suited.

**Figure 8:**
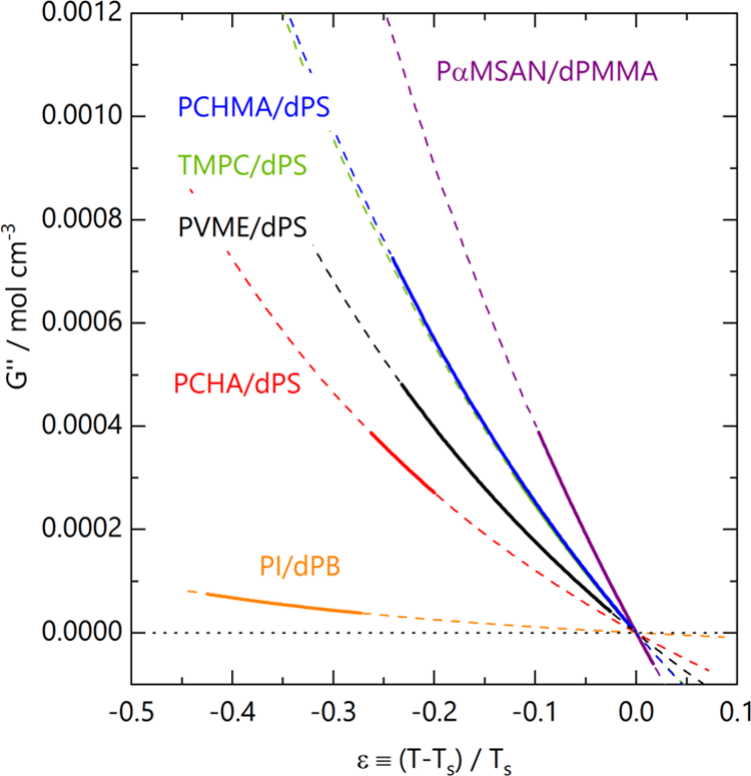
Comparison of *G*″(*T*) for a range of LCST blends alongside the current results for PCHMA/dPS (all ∼50/50 v/v compositions). ϵ is a reduced temperature describing the normalized proximity to *T*_s_. Solid lines correspond to *G*″ determined from SANS, and dashed lines are extrapolations outside of the measured temperature range. Reference data: PI/dPB (PI *M*_w_ = 115 kg mol^–1^, 70% cis units, dPB *M*_w_ = 275 kg mol^–1^) ref (39), PCHA/dPS (PCHA *M*_w_ = 465 kg mol^–1^, dPS *M*_w_ = 99 kg mol^–1^) ref (40), PVME/dPS (PVME *M*_w_ = 159 kg mol^–1^, dPS *M*_w_ = 195 kg mol^–1^) ref (41), TMPC/dPS (TMPC *M*_w_ = 54 kg mol^–1^, dPS *M*_w_ = 225 kg mol^–1^) ref (23), and PαMSAN/dPMMA (PαMSAN *M*_w_ = 122 kg mol^–1^, dPMMA *M*_w_ = 39.5 kg mol^–1^) ref (32).

We finally compare the magnitude of *G*″(*T*) for this blend with others determined by SANS for previously reported polymer blends in Figure 8. We select a few representative LCST systems, including poly(isoprene)/deuterated poly(butadiene) (PI/dPB),^39^ poly(cyclohexyl acrylate)/dPS (PCHA/dPS),^40^ poly(vinyl methyl ether)/deuterated poly(styrene) (PVME/dPS),^41^ tetramethyl bisphenol-A polycarbonate/deuterated poly(styrene) (TMPC/dPS),^23^ and poly(α-methylstyrene-*co*-acrylonitrile)/deuterated poly(methyl methacrylate) PαMSAN/dPMMA.^32,33^ As each blend generally exhibits a different spinodal temperature *T*_s_ and has thus been measured over a different temperature range, we rescale the temperature axis with respect to *T*_s_, employing a dimensionless quench depth ϵ ≡ (*T* – *T*_s_)/*T*_s_. An alternative scaling of temperature by the difference from the spinodal temperature alone (*T* – *T*_s_) is provided in SI Figure S8. This comparison places PCHMA/dPS among the most highly interacting blends in the literature, as defined by the steepness of *G*″ variation with the temperature near the spinodal. Such highly interacting systems respond strongly to modest changes in temperature and, when quenched into the unstable region, have the potential of yielding nanoscale demixed length scales relevant to a range of applications.^4^ This system exhibits a steeper temperature dependence of *G*″ than PVME/dPS, almost identical to TMPC/dPS and slightly lower than PαMSAN/dPMMA (whose phase behavior is, however, very sensitive to copolymer tacticity).^33^

## Conclusions

We have explored the thermodynamics of a highly interacting LCST PCHMA/dPS blend by SANS, supported by optical and atomic force microscopy, thermal gravimetric, and calorimetric measurements. The blend degrades rapidly at temperatures approaching the phase boundary, with spinodal *T*_s_ = 245.3 ± 0.9 °C from SANS near the critical composition, which is above the ceiling temperatures of both constituent polymers (>200 °C). Isothermal SANS measurements in the one-phase region are well described by the RPA, providing measurements of *G*″(*T*) (or equivalently χ̅/*v*_0_) and the segment length for PCHMA.

Using TOF-SANS at low wavelength resolution, we examine a series of temperature-jump experiments within the one-phase region. Instead of employing CHC, we find that the transient scattering profiles are well described by RPA, which we interpret as due to the high mobility *M* of this system at *T* ≫ *T*_g_, relative to the timescales of the *T*-jumps in our setup. In order to evaluate under what conditions a temperature jump can be considered ‘slow’ or ‘fast’ and thus whether RPA or CHC are the appropriate theoretical frameworks, we introduce an ‘equilibration time,’ *t*_e_, based on CHC theory and a ‘proximity’ criterion, σ, which we set at 0.05 (or 5%). This time *t*_e_ estimates the time interval to reach within σ of the equilibrium *S*(*q*) of the final temperature of the jump. It is reminiscent of the ‘early stage’ criterion for spinodal decomposition (albeit with a different origin) and of Binder and co-workers’^37^ study of ‘slow’ jumps during spinodal decomposition. With knowledge of *G*″(*T*), computing *t*_e_ as a function of *M* for jumps Δ*T* ≡ *T*_f_ – *T*_*i*_ permits a facile estimation of whether these can be considered slow or fast and thus whether RPA or CHC is appropriate.

The data acquired by both isothermal and temperature-jump experiments are in excellent agreement, yielding χ̅/*v*_0_ = 0.00115 – 0.591/*T* (with reference *v*_0_ ≡ 123.8 cm^3^ mol^–1^) and *G*″ = −0.00228 + 1.1821/*T* mol cm^–3^, and the segment length of *a*_PCHMA_ = 13.9 ± 0.6 Å. The LCST phase boundaries of this system are reasonably well described by Flory–Huggins theory (within measurement uncertainty) and this χ parameter, which differs from previous reports. Finally, comparison to other blends in terms of the reduced temperature ϵ places PCHMA/dPS blends among the most highly interacting (in terms of the steepness of the temperature dependence of *G*″) LCST systems reported in the literature, with (reasonably) accessible *T*_s_. Such systems have the potential to yield small demixed bicontinuous phase sizes upon a temperature jump into the spinodal region, which could be rendered accessible with greater polymer *M*_w_ and chemical stabilizers.

## Supporting Information Available

Summary of film preparation and properties employed in SANS measurements; configuration of monochromatic SANS film measurements; SANS data reduction & incoherent background; differential scanning calorimetry data of blends; Flory–Huggins description of optical cloud points; thermal gravimetric analysis; D33 Kratky analysis; blend composition dependence of scattering data; experimental temperature-jump profiles; estimation of equilibration timescales based on Cahn–Hilliard–Cook theory; and blend *G''* comparison (PDF)
