# Supplementary material for: Thermodynamics of Highly Interacting Blend PCHMA/dPS by TOF-SANS
Source: Macromolecules. 2023 Jul 13;56(14):5619–27. doi: 10.1021/acs.macromol.3c00511 (PMC10373520; doi:10.1021/acs.macromol.3c00511)
Supplement: Supplementary file 1 — ma3c00511_si_001.pdf [file ma3c00511_si_001.pdf]

Supplementary Information: Thermodynamics of Highly Interacting Blend PCHMA/dPS by TOF-SANS

William N. Sharratt,[†] Yutaka Aoki,[†] Dale Seddon,[†] Sebastian Pont,[†] Charles Dewhurst,[‡] Lionel Porcar,[‡] Nigel Clarke,[¶] and João T.Cabral[*,†]

†*Department of Chemical Engineering, Imperial College London, London SW7 2AZ, UK*

‡*Institut Laue Langevin, 71 Avenue des Martyrs, 38000 Grenoble, France*

¶*Department of Physics, The University of Sheffield, Sheffield, S10 2TN, UK*

E-mail: j.cabral@imperial.ac.uk

Phone: +44 207 594 5571

1. Summary of Films for SANS Measurements

A summary of the films measured with SANS in this study is provided in Table S1. The volume fraction of PCHMA in the films are calculated from the bulk densities of each component ($\rho_{PCHMA} = 1.10$ g cm^{-3} and $\rho_{dPS} = 1.12$ g cm^{-3}) and the mass fractions in the blend. The thickness are averages of multiple measurements across the film with a micrometer.

Table S1: Details of films measured by SANS: volume fraction of PCHMA in the film (ϕ_{PCHMA}), beamline, measurement/annealing temperature(s) (T), measured thicknesses (t_m), fitted thicknesses (t_f), measured transmission value(s) and background/incoherent scattering intensity value(s) (B).

ϕ_{PCHMA}	Beamline	T / °C	t_m / mm	t_f / mm	Transmission(s)	B / cm^{-1}
0.046	D22	150	0.074	0.150	0.9823	0.056
0.151	D22	150	0.081	0.120	0.9888	0.095
0.243	D22	150	0.373	0.116	0.9670	0.129
0.345	D22	150	0.278	0.086	0.9588	0.166
0.445	D22	150	0.139	0.029	0.9822	0.202
0.492	D22	150	0.070	0.032	0.9788	0.219
0.547	D22	150	0.066	0.047	0.9672	0.239
0.646	D22	150	0.053	0.045	0.9507	0.275
0.748	D22	150	0.051	0.040	0.9624	0.312
0.941	D22	150	0.074	0.067	0.9722	0.447
1	D22	150	0.662	0.662	0.7471	0.485
1	D22	150	0.270	0.270	0.8875	0.404
0.495	D33	120,200,240	0.956	0.956	0.7500,0.7219,0.7332	0.123,0.103,0.098
1	D33	100	0.080	0.080	0.9544	0.0374
1	D33	100	0.322	0.322	0.8838	0.448
1	D33	150	0.440	0.440	0.8110	0.443
1	D33	25,100,200	0.560	0.560	0.8397,0.8301,0.8105	−, −, −
1	D33	100	0.979	0.979	0.6552	0.495

2. Monochromatic SANS Film Measurements

Measurements of thin films with varying composition (ϕ_{PCHMA}) and fixed annealing temperature (150 °C) were carried out on the D22 diffractometer (ILL, Grenoble, France) with incident neutron wavelength $\lambda = 6$ Å, $\Delta\lambda/\lambda = 10\%$. and sample-to-detector distances of 1.4, 5 and 17 m yielding a Q-range of 0.0028-0.61 Å^{-1}.

3. SANS Data Reduction & Incoherent background

The incoherent scattering background of the polymer blend films, assuming ideal volume mixing, is expected to follow approximately the volume average of the background of the pure components:

$$I_{inc} = \phi_{dPS}S_{inc,dPS} + \phi_{PCHMA}S_{inc,PCHMA}. \tag{1}$$

where $S_{inc,dPS} \approx 0.04$ cm^{-1},[1] and $S_{inc,PCHMA}$ was measured on D22 with films of varying thickness, as detailed in Table S1. The coherent scattering signal was obtained by subtraction of the incoherent (or background) scattering. The background/incoherent values for films measured on D22 agree well with the expected incoherent scattering, while deviations are observed for TOF data acquired in D33. This is not unexpected given the complexities of energy-dependent incoherent scattering analysis, in particularly for hydrogen-rich and weakly scattering systems. Uncertainties in background (~ 0.1 cm^{-1}) are, however, of marginal importance to the value of $I(0)$ ($\sim 10 - 100$ cm^{-1}) and by definition G'' or $\bar{\chi}_{12}$.

Given the low film thickness ($\sim$ 50-400 μm) of the samples measured on D22, and associated uncertainty related to the uniformity of cross section and fraction of film within the neutron beam illuminated area, we carefully checked for the self-consistency of the data in terms of thickness, transmission and incoherent background. In monochromatic SANS mode, data reduction and calibration follows:

$$I_s(q) = K_{abs}\left(\frac{I_{s+c}(q) - I_b}{t_{s+c}T_{s+c}} - \frac{I_c(q) - I_b}{t_cT_c}\right) \tag{2}$$

where K_{abs} is a calibration constant, to convert the data into absolute [cm−1] units and

measured with direct beam flux and solid angle. I_i, t_i and T_i refer to the scattering, thickness and transmission of species i, respectively. The subscripts s, $s+c$, c and b refer to the 'sample', 'sample and cell', 'cell' and 'cadmium blank/background'. Our initial reduction of the D22 data with measured films thicknesses and measured transmissions yielded some inconsistencies. For instance, in some samples the incoherent scattering/background and the transmission values were not consistent for the known blend composition. We have therefore considered the sample transmission (T) in terms of the Beer-Lambert law,

$$T = \exp(-\mu t) \tag{3}$$

where μ is the neutron attenuation coefficient of the film material (mm^{-1}) and t is the film thickness (mm). The neutron attenuation coefficient is related to the total cross section of the scattering sample and therefore could be, in the first approximation, estimated here as a linear combination of the attenuation coefficients of dPS (μ_{dPS})and PCHMA (μ_{PCHMA}):

$$\mu_{film} = \phi_{dPS}\mu_{dPS} + \phi_{PCHMA}\mu_{PCHMA} \tag{4}$$

where ϕ_i is the volume fraction of either dPS or PCHMA in the film. From the transmission values of thicker PCHMA films measured on D33, shown in Table S1, we calculate that $\mu_{PCHMA} = 0.441 \pm 0.001\ \mathrm{mm}^{-1}$ and impose that $\mu_{dPS} = 0.084\ \mathrm{mm}^{-1}$ from previous measurements.[1] Given our known volume fractions of dPS and PCHMA in the films, and from Equation 4, we can calculate the expected attenuation coefficient for each film. The intensity of the films' incoherent signal is given in Equation 1, where $S_{inc,dPS} \sim 0.04\ \mathrm{cm}^{-1}$ is known,[1] and a narrow range for $S_{inc,PCHMA}$ is determined from measurements of two thicknesses of PCHMA films on D22. The incoherent value of blend films is expected to follow monotonically with ϕ_{PCHMA} and, for pure PCHMA films, with sample thickness. We have therefore estimated the sample thickness, from the transmission value and attenuation coefficient, and then adjusted the thickness by a small factor to yield an incoherent background proportional to ϕ_{PCHMA} for all samples - $S_{inc,PCHMA} \sim 0.514\ \mathrm{cm}^{-1}$. We independently checked our approach by comparing the value of G'' and $\bar{\chi}/v_0$ for the 50/50 blend measured on D22 with that in the main paper in Figure S1. Both the G'' and $\bar{\chi}/v_0$ values obtained

for the blend measured on D22 are consistent. At the lowest temperature, near T_g, there is greater uncertainty which is attributed to slow quenching and incomplete equilibration of the fluctuation spectrum.

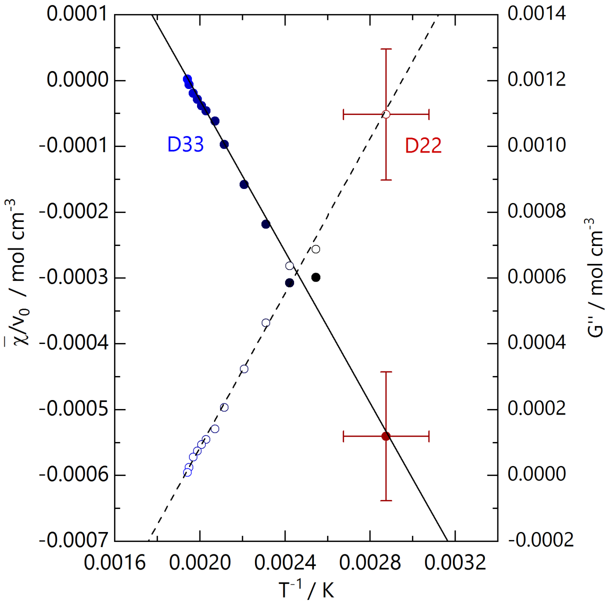

Figure S1: Comparison of $\bar{\chi}/v_0$ and G'' for 50/50 blend films on both D22 (red circles) and D33 (black to blue circles). Horizontal error bars are the uncertainty in $\bar{\chi}/v_0$ and G'' propagated into the expected temperature of blend.

4. Differential Scanning Calorimetry

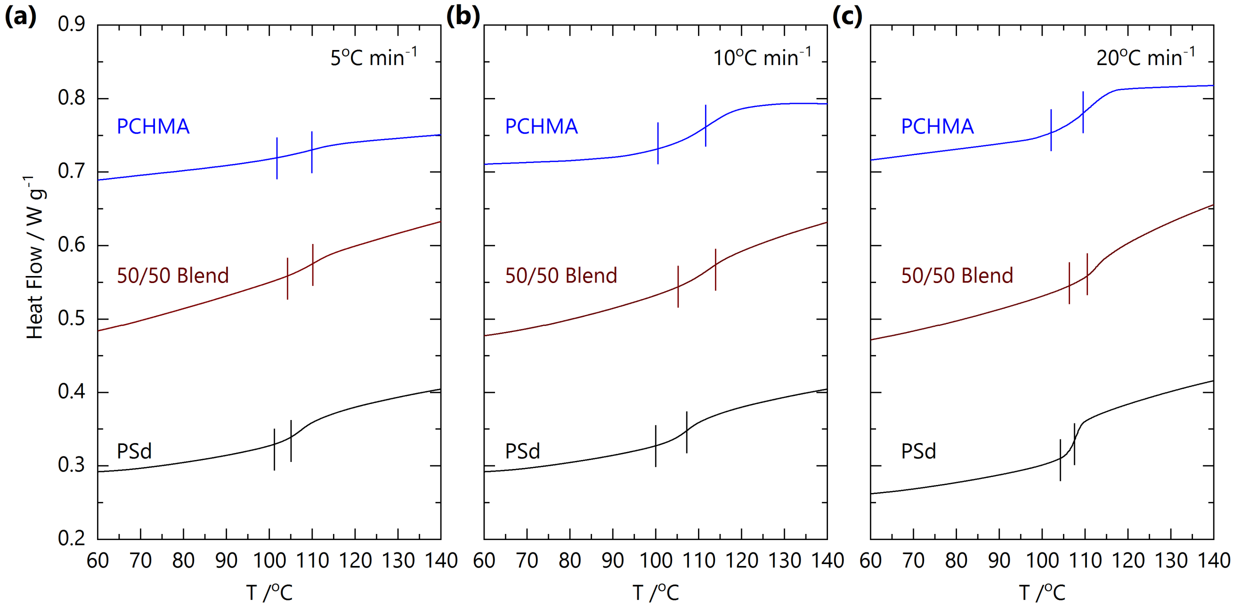

Figure S2: DSC thermograms of PCHMA, dPS and 50/50 w/w blend measured at 5°C min^{-1} (a) 10°C min^{-1} (b) and 20°C min^{-1} (c). Curves are shifted vertically for comparison. Glass transitions are apparent in the neat polymers and blend with onsets shifted to higher temperatures as the temperature ramp rate is increased.

Table S2: Onset and onset midpoints of DSC thermograms measured at 5, 10 and 20 °C min^{-1} for PCHMA, dPS and PCHMA/dPS blends.

PCHMA		
Rate / °C min^{-1}	Onset / °C	Onset Midpoint / °C
5	99.32	110.03
10	101.64	112.29
20	103.17	110.51
dPS		
Rate / °C min^{-1}	Onset / °C	Onset Midpoint / °C
5	100.46	105.84
10	100.57	107.40
20	104.90	107.82
Blend (50/50 w/w)		
Rate / °C min^{-1}	Onset / °C	Onset Midpoint / °C
5	105.45	110.54
10	106.46	114.34
20	109.22	112.62

5. Flory-Huggins Description of Optical Cloud Point

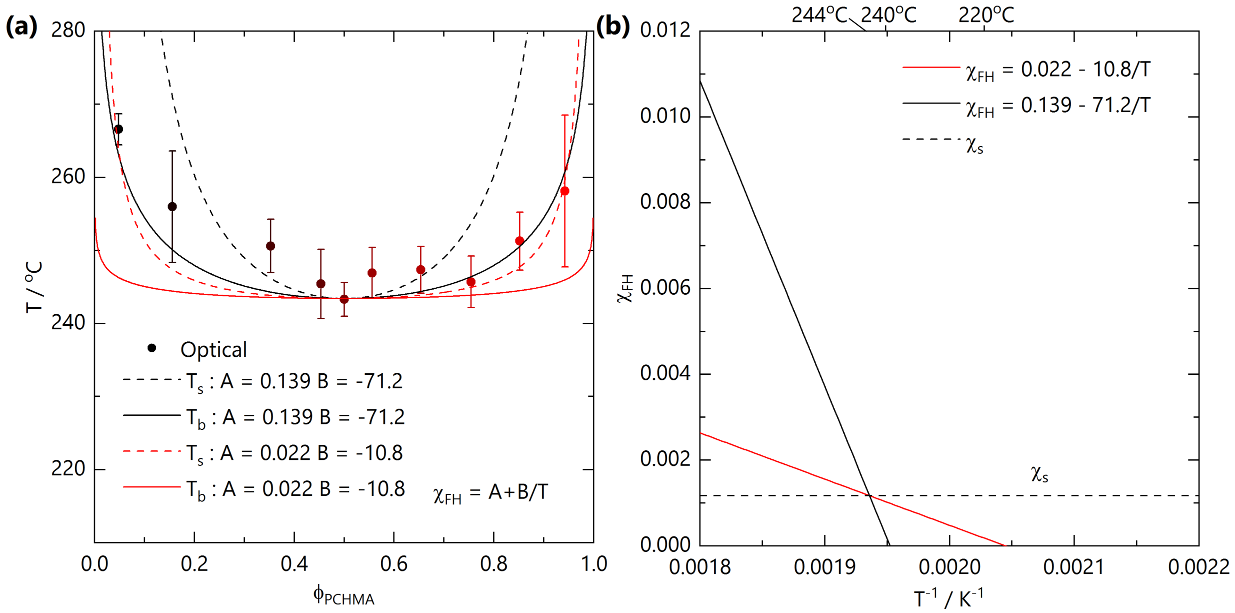

Figure S3: (a) Comparison of spinodal and binodal lines (temperatures) from Flory-Huggins theory with experimental optical cloud point measurements. The Flory-Huggins interaction parameters $\chi_F H$ is given by a reciprocal relationship with temperature $\chi_{FH} = A + B/T$. Two cases are compared: the relationship derived from a combination of our optical measurements and corroborated by SANS, with $A = 0.139$ and $B = -71.2$, and $A = 0.022$ and $B = -10.8$ taken from Friedrich *et al.*[2] (b) Temperature dependence of χ_{FH} for both cases. Despite the large difference in B, intersection with χ_s in each case yields $T_s \sim 0.00193\ \mathrm{K}^{-1} \sim 244$°C. Temperatures corresponding to $1/T$ values are indicated on the top axis.

6. Thermal Gravimetric Analysis

The thermal degradation of 50/50 w/w blend films was assessed by Thermal Gravimetric Analysis (TGA) on a NZ STA Jupyter instrument. Samples were rapidly (50 °C min^{-1}) heated from above the T_g of the film to 240°C and held isothermally for 60 minutes. This simple ramp and isothermal hold was selected to estimate the extent of degradation approaching the phase boundary of the blend. Figure S1 shows the temperature profile and percentage mass loss of a blend film. Once a temperature of 240 °C is reached, the blend is stable for several minutes and after $\sim$ 5 min, the blend has lost $\sim$ 1% mass. The presence of oligomeric or monomeric species can impact blend miscibility and thermodynamics and thus

shift phase boundaries. Depolymerisation could also reduce chain dimensions and increase polydispersity, which would affect the high-q scattering and apparent segment length.

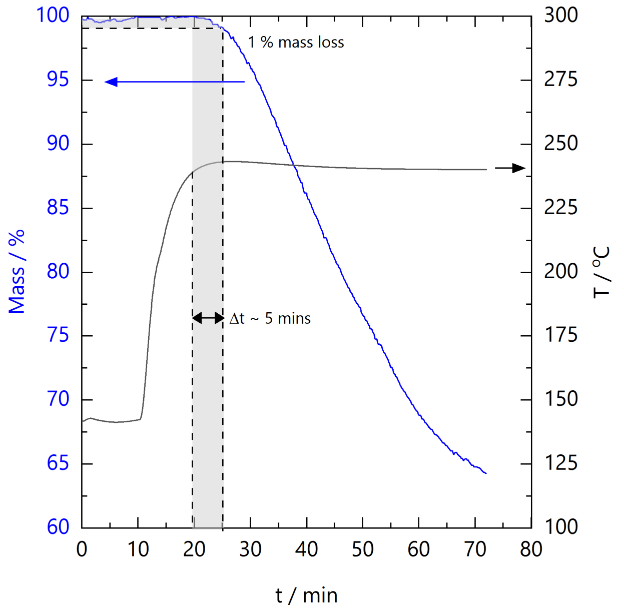

Figure S4: Mass loss of 50/50 w/w blend film upon heating at 50 °C min^{-1} (blue, left axis). The temperature profile during the measurement is also indicated (black, right axis). Vertical dashed lines indicate the time at which the blend film first reaches 240°C ($\sim$ 20 min) and once the film has lost 1% mass ($\sim$ 25 min), which is indicated by the horizontal dashed line. These areas are also shaded. Δt is approximately the time in which the film is stable at these temperatures, setting therefore a time limit for SANS measurements.

7. D33 Kratky Analysis

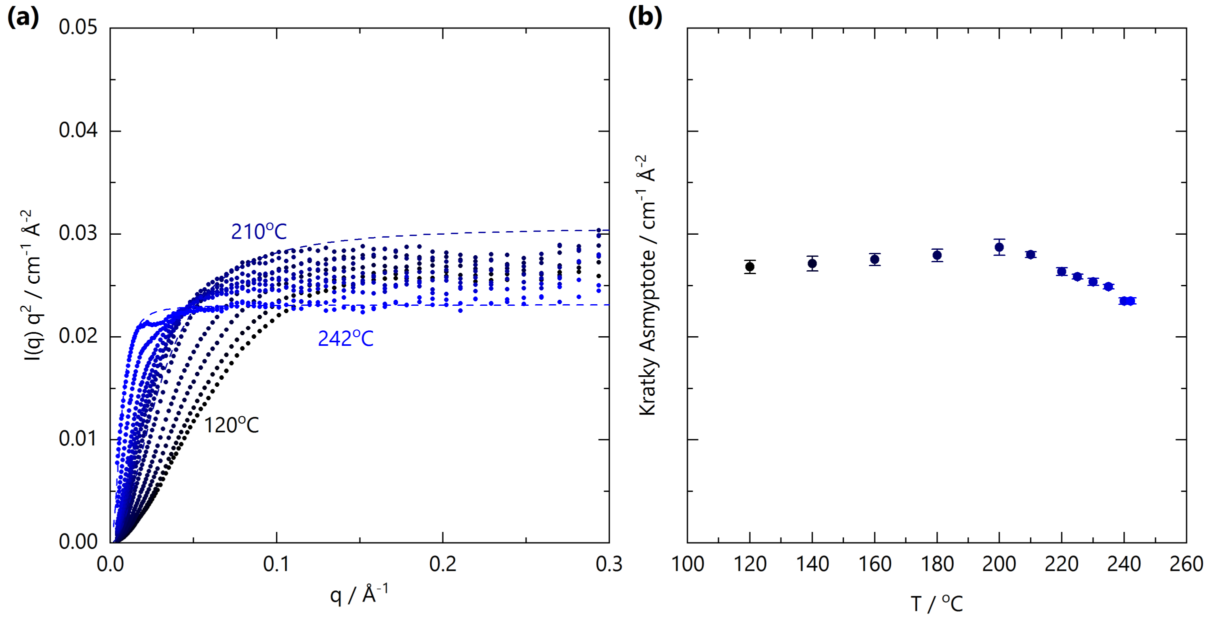

Figure S5: (a) Kratky plot of data in main paper Figure 3. RPA envelopes to illustrate lower and upper bounds of the Kratky asymptote are shown as dotted lines. (b) Asymptotic Kratky values as a function of temperature. Values lie in the range 0.0235 - 0.0280 cm^{-1} Å^{-2} and yield an average PCHMA segment length of 13.9 ± 0.6 Å.

8. Blend Composition Dependence

SANS curves and RPA fits to blend films with $\phi_{PCHMA} = 0.05 - 0.95$ are shown in Figure S6a. The RPA describes reasonably well all data for $\phi_{PCHMA} = 0.15 - 0.95$ for $0.01 < q < 0.3$ Å^{-1}. At low-q, excess forward scattering is observed which has been attributed to microvoids and trapped air.[3] The low-q scattering appears more prevalent for low ϕ_{PCHMA} blends, possibly due to greater void contrast. A plateau can still be observed before the low-q upturn in each case an an $I(0)$ determined. The RPA provides yields good agreement with the data, except for extreme concentrations, namely for $\phi_{PCHMA} = 0.05$ where the apparent PCHMA segment length is < 5 Å. Similarly, for $\phi_{PCHMA} = 0.95$, the data are not well modelled by RPA. Deviations of the RPA at extreme compositions are expected as the mean-field approximation eventually breaks down.

Figure S6b and S6c show the extracted G'' and $\bar{\chi}/v_0$ from RPA fits, respectively. In both cases the dashed lines, indicate a shallow parabolic dependence of the blend thermodynamics and interactions with ϕ_{PCHMA}. $G''(T \sim T_g) = -3.69 \times 10^{-4} + 0.0039\phi_{PCHMA} - 0.0032\phi_{PCHMA}^2$ and $\bar{\chi}/v_0(T \sim T_g) = 2.07 \times 10^{-4} - 0.0020\phi_{PCHMA} + 0.0017\phi_{PCHMA}^2$. For $\phi_{PCHMA} > 0.45$ both G'' and $\bar{\chi}/v_0$ can effectively be considered constant within error. The optical cloud point data are adequately modelled by Flory-Huggins theory assuming a composition independent χ.

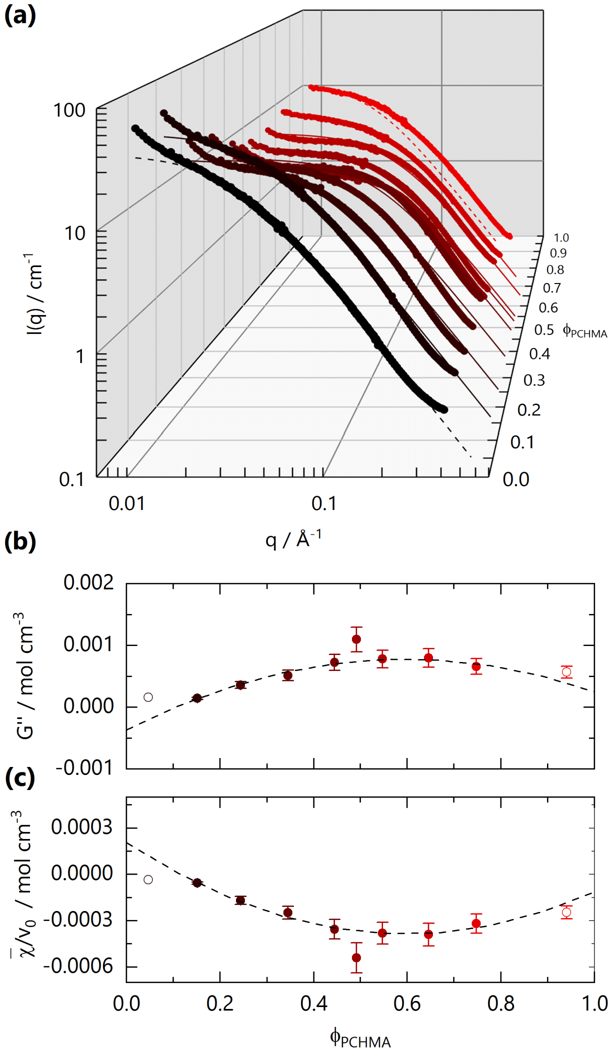

Figure S6: (a) Coherent scattering profiles from blend films measured on D22 with ϕ_{PCHMA} = 0.05, 0.15, 0.25, 0.35, 0.45, 0.50, 0.55, 0.65, 0.75 and 0.95 (black to red circles). RPA fits (solid lines) provide good agreement with the data, except for the extreme compositions indicated with dashed lines. (b) Composition dependence of G'' determined from application of the Ornstein-Zernike equation to data in (a). G'' has a maximum for the 50/50 blend and roughly follows a parabolic dependence on composition. (c) Composition dependence of $\bar{\chi}/v_0$ from RPA fits in (a). A minima is observed for the 50/50 blend and the composition dependence appears, again, parabolic in nature. In both (b) and (c) dashed lines are guides to the eye.

9. Experimental Temperature Jump Profiles

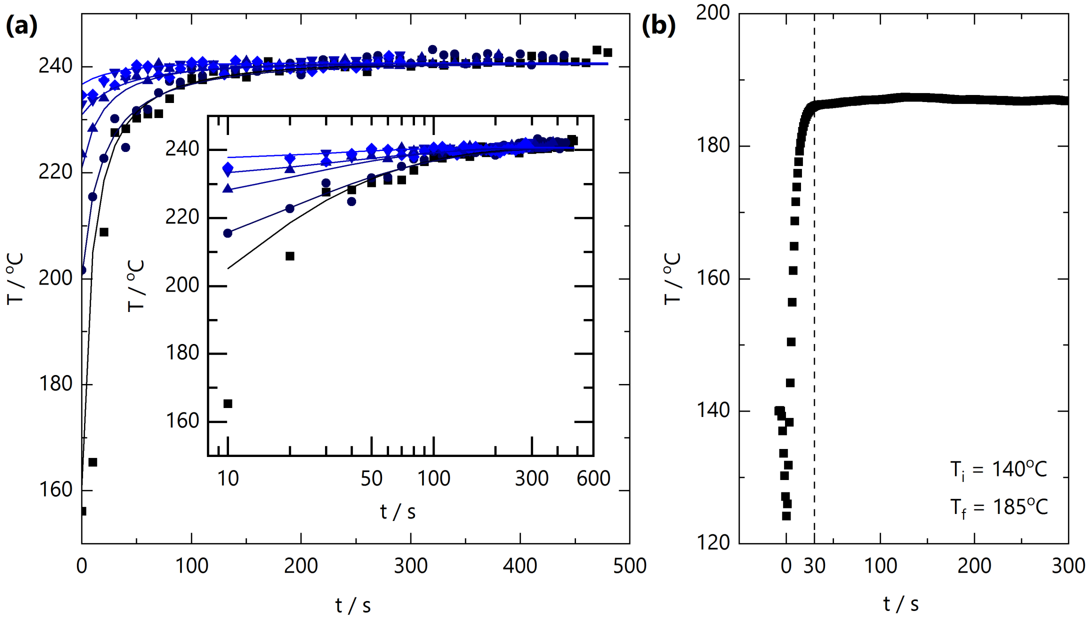

Figure S7: (a) Temperature profiles inferred from RPA fits to data in Figure 6d. Symbol colours and shape correspond to T_i values in Figure 6d. (Inset) Temperature profiles shown on a logarithmic time scale which highlight the long (> 60 s) apparent equilibration times t_e at $T_f = 240$°C. (b) Measured temperature profile for a jump from $T_i = 140$°C to $T_f = 185$°C. The negative times indicate the movement of the sample from preheating block which is accompanied by an initial dip in the measured temperature.

10. Estimation of Equilibration Timescales Based on Cahn-Hilliard-Cook Theory

Cahn-Hilliard-Cook (CHC) theory considers the effects of thermal fluctuations in the scattering from binary blends. Following Binder,[4] Strobl,[5] deGennes[6] and Pincus,[7] the evolution of $S(q,t)$ following a temperature quench can be expressed as:

$$\frac{\partial S(q,t)}{\partial t} = 2Mq^2\left(\frac{S(q,t)}{S_f(q)} - 1\right) \tag{5}$$

where $S_f(q)$ is the 'final' scattering from the blend at the end temperature T_f of the jump, well described by RPA (note that within the spinodal region, where G'' is negative, this function becomes negative over part of the q-range and is thus not observable experimentally). Eq. 5 can be solved to give:

$$S(q,t) = \Big(S(q,0) - S_f(q)\Big)e^{2R(q)t} + S_f(q) \tag{6}$$

where $S_i(q) \equiv S(q,t=0)$ is the 'initial' scattering profile, at the start temperature of the jump, T_i. The scattering profile $S(q,t)$ thus evolves asymptotically from $S_i(q)$ to $S_f(q)$ over a period of time, that depends on a range of system parameters. In order to estimate the time required for $S(q,t)$ to 'nearly' reach $S_f(q)$ characteristic of T_f, we introduce a 'proximity parameter' σ (with $0 \leq \sigma \leq 1$) such that:

$$S(q,t_e) = S_f(q)(1-\sigma) \tag{7}$$

and thus an 'equilibration time' t_e corresponding to reaching within σ of the final profile, for instance $\sigma \equiv$0.05 or 0.10, i.e. 5-10% of the final scattering profile (commensurate with measurement uncertainty, for short acquisition times). Combining Eq. 7 and Eq. 6 yields,

$$S_f(q)(1-\sigma) = \Big(S_i(q) - S_f(q)\Big)e^{2R(q)t_e} + S_f(q)$$

which can be rearranged to yield an expression of the equilibration timescale,

$$t_e \simeq \frac{\ln\left[\frac{\sigma S_f(q)}{S_f(q)-S_i(q)}\right]}{2R(q)} \tag{8}$$

The blend structure factor $S(q)$ can be expressed according to de Gennes's Random Phase Approximation (RPA) which, at low scattering angles is well-approximated by,

$$\frac{1}{S(q)} = \frac{1}{S(0)} + 2kq^2 \tag{9}$$

where k is the so-called 'square gradient term' of the free energy functional. The growth rate $R(q)$ according to CHC theory as

$$R(q) = -Mq^2(G'' + 2kq^2) \equiv -Mq^2/S_f(q) \tag{10}$$

M is a 'diffusional mobility' parameter.

In the RPA framework,

$$k = 1/6\left(R_{g1}^2/(\phi_1 v_1 N_1) + R_{g2}^2/(\phi_2 v_2 N_2)\right)$$

The RPA formulation emphasises that the forward scattering intensity yields $S(q \to 0) \equiv 1/G''$. Since concentration fluctuations equilibrate slowest at low q, we approximate $S(q)$ in Eq. 8 in this limit, yielding

$$t_e \approx \frac{\ln\left[\sigma\frac{1/G''_f}{1/G''_f - 1/G''_i}\right]}{-2Mq^2G''_f} = \frac{\ln\left[\sigma\frac{G''_i}{G''_i - G''_f}\right]}{-2Mq^2G''_f} \tag{11}$$

which can be alternatively expressed in terms of the corresponding χ parameter, noting that $G''(T) \equiv 2(\chi_s - \chi_T)/v$, as:

$$t_e \approx \frac{\ln\left[\sigma\frac{(\chi_s-\chi_i)}{\chi_f-\chi_i}\right]v}{4Mq^2(\chi_f - \chi_s)} \tag{12}$$

in order to estimate t_e, we evaluate the expressions above at the lowest experimentally

measured q value, i.e. $q_{min} \approx 0.003$ Å^{-1}, yielding the longest t_e within the measured window. Measurement times longer than t_e within a ΔT jump should reach equilibrium of the concentration fluctuation spectrum, and thus yield a good approximation to $S(q)$ at the corresponding temperature T. Based on the above approximations, we compute a map of equilibration times t_e as a function of mobility M and quench depth ($\Delta G'' \equiv G''_f - G''_i$, or $\Delta\chi \equiv \chi_f - \chi_i$), and for a given interaction strength (G''_f or $\chi_s - \chi_f$).

11. Blend G'' Comparison

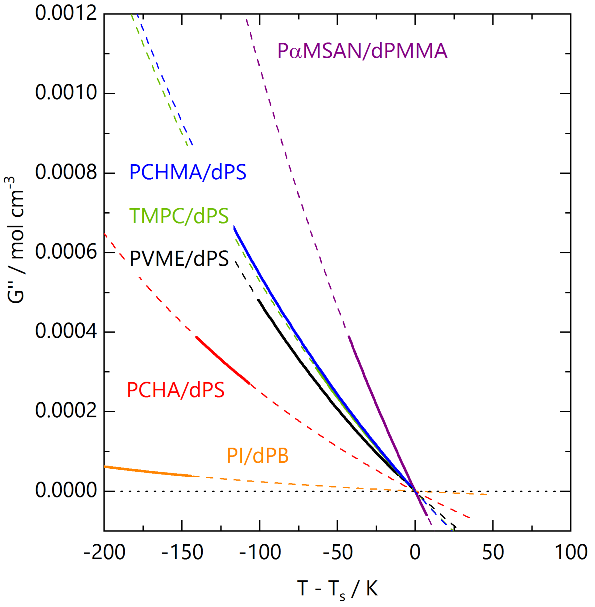

Figure S8: Comparison of G'' from previous SANS reports on polymer blends, alongside PCHMA/dPS blend studied here (all $\sim$ 50/50 v/v compositions). The temperature axis is scaled relative to the spinodal temperature T_s for each blend. Solid lines are G'' values determined from SANS and dashed lines are extrapolations outside of the measured temperature range. Reference data: PI/dPB (PI M_w = 115 kg mol^{-1}, 70% cis units, dPB M_w = 275 kg mol^{-1}) Ref 8, PCHA/dPS (PCHA M_w = 465 kg mol^{-1}, dPS M_w = 99 kg mol^{-1}) Ref 9, PVME/dPS (PVME M_w = 159 kg mol^{-1}, dPS M_w = 195 kg mol^{-1}) Ref 10, TMPC/dPS (TMPC M_w = 54 kg mol^{-1}, dPS M_w = 225 kg mol^{-1}) Ref 1 and PαMSAN/dPMMA (PαMSAN M_w = 122 kg mol^{-1}, dPMMA M_w = 39.5 kg mol^{-1}) Ref 11.

References

1. Cabral, J. T.; Higgins, J. S. Small Angle Neutron Scattering from the Highly Interacting Polymer Mixture TMPC/PSd: No Evidence of Spatially Dependent χ Parameter. *Macromolecules* **2009**, *42*, 9528–9536.

2. Friedrich, C.; Schwarzwälder, C.; Riemann, R.-E. Rheological and thermodynamic study of the miscible blend poly styrene/poly(cyclohexyl methacrylate). *Polymer* **1996**, *37*, 2499 – 2507.

3. Xie, S.; Zhang, B.; Mao, Y.; He, L.; Hong, K.; Bates, F. S.; Lodge, T. P. Influence of Added Salt on Chain Conformations in Poly(ethylene oxide) Melts: SANS Analysis with Complications. *Macromolecules* **2020**, *53*, 7141–7149.

4. Binder, K. Collective diffusion, nucleation, and spinodal decomposition in polymer mixtures. *The Journal of Chemical Physics* **1983**, *79*, 6387–6409.

5. Strobl, G. R. Structure evolution during spinodal decomposition of polymer blends. *Macromolecules* **1985**, *18*, 558–563.

6. Gennes, P. G. d. Dynamics of fluctuations and spinodal decomposition in polymer blends. *The Journal of Chemical Physics* **1980**, *72*, 4756–4763.

7. Pincus, P. Dynamics of fluctuations and spinodal decomposition in polymer blends. II. *The Journal of Chemical Physics* **1981**, *75*, 1996–2000.

8. Tomlin, D. W.; Roland, C. M. Negative excess enthalpy in a van der Waals polymer mixture. *Macromolecules* **1992**, *25*, 2994–2996.

9. Schubert, D. W.; Abetz, V.; Stamm, M.; Hack, T.; Siol, W. Composition and Temperature Dependence of the Segmental Interaction Parameter in Statistical Copolymer/Homopolymer Blends. *Macromolecules* **1995**, *28*, 2519–2525.

10. Hammouda, B.; Briber, R. M.; Bauer, B. J. Small angle neutron scattering from deuterated polystyrene/poly(vinylmethyl ether)/protonated polystyrene ternary polymer blends. *Polymer* **1992**, *33*, 1785 – 1787.

11. Aoki, Y.; Wang, H.; Sharratt, W.; Dalgliesh, R. M.; Higgins, J. S.; Cabral, J. T. Small Angle Neutron Scattering Study of the Thermodynamics of Highly Interacting PαMSAN/dPMMA Blends. *Macromolecules* **2019**, *52*, 1112–1124.
